# Free water imaging unravels unique patterns of longitudinal structural brain changes in Parkinson’s disease subtypes

**DOI:** 10.3389/fneur.2023.1278065

**Published:** 2023-10-30

**Authors:** Abigail E. Bower, Sophia J. Crisomia, Jae Woo Chung, Justin P. Martello, Roxana G. Burciu

**Affiliations:** ^1^Department of Kinesiology and Applied Physiology, University of Delaware, Newark, DE, United States; ^2^Department of Neurology, University of Minnesota, Minneapolis, MN, United States; ^3^Department of Neurosciences, Christiana Care Health System, Newark, DE, United States

**Keywords:** Parkinson’s disease, TD, PIGD, DTI, progression

## Abstract

**Background:**

Research shows that individuals with Parkinson’s disease (PD) who have a postural instability and gait difficulties (PIGD) subtype have a faster disease progression compared to those with a tremor dominant (TD) subtype. Nevertheless, our understanding of the structural brain changes contributing to these clinical differences remains limited, primarily because many brain imaging techniques are only capable of detecting changes in the later stages of the disease.

**Objective:**

Free water (FW) has emerged as a robust progression marker in several studies, showing increased values in the posterior substantia nigra that predict symptom worsening. Here, we examined longitudinal FW changes in TD and PIGD across multiple brain regions.

**Methods:**

Participants were TD and PIGD enrolled in the Parkinson’s Progression Marker Initiative (PPMI) study who underwent diffusion MRI at baseline and 2 years later. FW changes were quantified for regions of interest (ROI) within the basal ganglia, thalamus, brainstem, and cerebellum.

**Results:**

Baseline FW in all ROIs did not differ between groups. Over 2 years, PIGD had a greater percentage increase in FW in the putamen, globus pallidus, and cerebellar lobule V. A logistic regression model incorporating percent change in motor scores and FW in these brain regions achieved 91.4% accuracy in discriminating TD and PIGD, surpassing models based solely on clinical measures (74.3%) or imaging (76.1%).

**Conclusion:**

The results further suggest the use of FW to study disease progression in PD and provide insight into the differential course of brain changes in early-stage PD subtypes.

## Highlights

- *De novo* PD with a PIGD subtype had a more rapid progression of motor symptoms over 2 years compared to PD with a TD subtype.
- PIGD exhibited a greater increase in free water (FW) over a 2 years period compared with TD within the putamen, globus pallidus, and cerebellar lobule V.
- The inclusion of the 2 years percent change in motor symptoms and FW levels within the putamen, globus pallidus, and cerebellar lobule V in a predictive model aimed to distinguish PD subtypes yielded a classification accuracy superior to that derived from models using only clinical or imaging measures. This finding remains to be validated in future, larger-scale studies.

## 1. Introduction

Parkinson’s disease (PD) is a complex neurodegenerative disease that is increasingly recognized as being heterogeneous in symptom presentation and progression (1–5). Currently, both the clinical and research communities acknowledge two distinct motor subtypes: tremor dominant (TD) and posture instability and gait difficulties (PIGD) subtypes (6–9). This classification is determined by the scores on specific items from Parts II and III of the Movement Disorder Society Unified Parkinson’s Disease Rating Scale (MDS-UPDRS) (10) and considers the prominence of tremor symptoms versus gait-related symptoms. Prior work revealed that PIGD have a greater burden of non-motor symptoms, including cognitive difficulties and dementia, (11–13) and autonomic dysfunction (14, 15). Regarding the progression of motor symptoms, individuals with a PIGD subtype have a faster progression compared to those with a TD subtype (9, 16).

Our understanding of the brain changes that contribute to these clinical differences is still in its early stages. Neuroimaging techniques, particularly those relying on magnetic resonance imaging (MRI), have demonstrated significant value in investigating structural changes in PD, and more recently in its various subtypes (17, 18). Prior studies found that compared to TD, moderate PIGD presented with more widespread structural changes (e.g., atrophy) within the prefrontal, parietal, and temporal brain regions (19, 20). However, it is worth noting that many MRI-based volumetric techniques tend to identify structural brain changes in PD during the later stages of the disease or in PD with dementia, and have reduced sensitivity to structural brain changes occurring in the early stages (21). Also, while conventional structural MRI techniques including voxel-based morphometry (VBM) are sensitive to cortical changes, they are not as optimal for detecting structural changes in some of the basal ganglia nuclei involved in the pathophysiology of the disease. This is because these techniques rely on the contrast in the image to identify brain structures, and the conventional T1-weighted contrast for nuclei such as the globus pallidus (GP), subthalamic nucleus (STN), and substantia nigra is poor (22). As a result, this can lead to inaccuracies or partial segmentations, along with difficulties in interpreting group differences. Moreover, the development and validation of robust T1-weighted-based progression biomarkers in PD, particularly for investigating longitudinal brain changes in its subtypes, are hindered by the increased susceptibility of these measures to scanner artifacts, which can impact the segmentation process.

To the best of our knowledge, no study has assessed the progression of structural brain changes in PD subtypes. This represents an important area of research that has yet to be fully explored. The variability in symptom presentation and progression of PD subtypes poses a significant challenge in clinical trials designed to evaluate potential disease-modifying drugs. Specifically, a drug’s effectiveness and safety may vary between PD subtypes. Therefore, gaining insight into the rate of disease progression in TD and PIGD subtypes could greatly enhance the design of clinical trials. Considering that disease-modifying drug candidates are intended to halt or slow the progression of neurodegeneration during the initial stages of the disease, it is imperative to gain deeper insights into the trajectory of brain changes in TD and PIGD during this critical period. This entails studying the progression of structural brain changes in PD subtypes using a PD progression marker sensitive to early alterations in brain structure.

Such a progression marker that is sensitive to early-stage microstructural brain changes in PD is free water (FW) (21). FW is a measure derived from diffusion tensor imaging (DTI) that assesses the presence of extracellular fluid in brain tissue (23, 24). In neurodegenerative diseases such as PD, water molecules may diffuse more freely due to increased extracellular spaces (25). Previous single-and multi-site studies utilizing FW in PD have established that when compared to healthy older adults, patients with PD have elevated FW in the posterior substantia nigra (PSN) (26–28). FW values in the PSN were correlated cross-sectionally with the severity of motor symptoms based on the MDS-UPDRS-III (26), DTBZ striatal binding (29), and increased longitudinally over 1 year, 2, and 4 years, with consistent longitudinal observations across multiple MRI platforms (26, 27). In conjunction with FW values in other brain regions, this approach discriminated PD from atypical parkinsonian syndromes like progressive supranuclear palsy (PSP) and multiple system atrophy (MSA) with high sensitivity and specificity in single- and multi-site studies (28, 30). Finally, in a large study involving drug naïve individuals with a recent PD diagnosis recruited as part of the Parkinson’s Progressive Marker Initiative (PPMI) study, a key finding was that short-term increases in FW in PSN were associated with long-term changes in motor symptoms (i.e., worsening of symptoms at the 4 years mark) (27). Collectively, these findings strongly advocate for the utility of FW in monitoring the progression of structural brain changes in TD and PIGD subtypes.

Therefore, the aim of this study was to leverage the capabilities of FW imaging to examine differences in the long-term structural brain changes between TD and PIGD from the PPMI study. This investigation included an examination of 2 years changes in FW in several brain regions important for motor control across the basal ganglia, thalamus, brainstem, and cerebellum. Importantly, the two PD groups had a stable subtype and did not differ significantly at baseline on age, sex, severity of motor symptoms, cognitive status, and FW measures across all regions of interest (ROIs). Due to an observed disparity in medication efficacy between TD and PIGD (31–33), it is plausible that PIGD may exhibit brain changes beyond the basal ganglia, in non-dopaminergic structures. Drawing upon previous observations concerning treatment response and prevailing motor symptoms, we hypothesized that compared to TD, PIGD will have greater increases in FW over the two-year period within the basal ganglia (including PSN) and the cerebellum and that this change will be accompanied by a faster rate of progression of motor symptoms.

## 2. Methods

### 2.1. PPMI participants

The data included in this study was obtained from the Parkinson’s Progressive Marker Initiative (PPMI; PPMI 1.0 protocol; https://www.ppmi-info.org/) between May and June of 2023. The PPMI study is a multi-site longitudinal study of PD, launched in 2010 by the Michael J. Fox Foundation, and designed to facilitate the discovery of diagnostic and progression biomarkers of PD that can aid in the development of innovative and possibly disease-modifying treatments (34, 35). The PPMI study provides open access to clinical, imaging, biospecimen, and genetic data collected from early-stage PD who were drug naïve at enrollment and had a clinical diagnosis confirmed by a dopamine transporter scan (DaTscan) (34, 35). The PPMI study obtained approval from the Institutional Review Board at each participating site, and all participants provided informed consent.

Given the purpose of our study to investigate the progression of structural brain changes in PD subtypes, we conducted an analysis of clinical and diffusion MRI data collected at baseline and after 2 years, specifically comparing data of PD with a TD subtype to PD with a PIGD subtype.

Participants were included in this study if they had: (1) a stable diagnosis of idiopathic PD (36), (2) a stable clinical subtype at baseline and year 2 determined based on a widely used formula applied to the Movement Disorders Society Unified Parkinson’s Disease Rating Scale (MDS-UPDRS) items scored off antiparkinsonian medication (9), and (3) diffusion MRI data using 64 directions available at these two time points. Additionally, participants were required to have at least a baseline motor severity score derived from part III of the MDS-UPDRS obtained off antiparkinsonian medication. It is important to highlight that due to potential variability in the clinical subtyping during the initial stages of the disease, we assessed the stability of the TD/PIGD subtypes over a 4 years follow-up in those with available data. However, since a significant number of PIGD patients had clinical scores but lacked imaging data at the 4 years mark, our longitudinal analysis focused on examining clinical and brain changes within a 2 years timeframe.

The following comorbidities were considered exclusion criteria: movement disorders other than PD, a history of cardiovascular diseases including stroke and transient ischemic attack, neuropsychiatric conditions, dementia, degenerative disk disorders, or a history of cancer requiring chemotherapy and/or radiation. Upon applying these inclusion and exclusion criteria, we identified a primary cohort (*Cohort 1*) of 46 PD with brain imaging available at each timepoint: 28 TD and 18 PIGD. The secondary cohort (*Cohort 2*) was a subset of Cohort 1 and consisted of PD with an MDS-UPDRS-III off-medication score at each time point: 23 TD and 12 PIGD. The role of this second cohort is explained in detail in the statistical analysis section. Importantly, in both cohorts, the two PD groups were well-matched at baseline (i.e., no statistically significant differences between groups) on the following measures: age, sex, severity of motor symptoms based on the MDS-UPDRS-III score off-medication and cognitive status as assessed by the Montreal Cognitive Assessment (MoCA) (37) (Table 1).

**Table 1:** Participant characteristics at baseline for PD in Cohorts 1 and 2.

Variable	COHORT 1		Value of *p*	COHORT 2		Value of *p*
	TD	PIGD		TD	PIGD	
Sample Size (N)	28	18	n/a	23	12	n/a
Age (Y)	62.11 (8.91)	62.84 (8.40)	0.781	61.77 (8.77)	64.98 (7.81)	0.295
Sex ♂ ♀	13 ♂ 15 ♀	13 ♂ 5 ♀	0.085	11 ♂ 12 ♀	8 ♂ 4 ♀	0.288
Baseline Hoehn & Yahr Stage	1.50 (0.51)	1.83 (0.51)	n/a	1.48 (0.51)	1.92 (0.51)	n/a
Baseline Total MDS-UPDRS-III	21.29 (8.03)	23.39 (9.26)	0.457†	21.83 (8.10)	25.33 (9.55)	0.296†
Baseline MDS-UPDRS-III - Bradykinesia	9.25 (4.28)	11.50 (6.18)	0.329†	9.17 (4.22)	11.58 (6.54)	0.400†
Baseline MDS-UPDRS-III - Rest Tremor	4.46 (2.03)	0.61 (1.24)	<0.001†	4.69 (2.08)	0.91 (1.44)	<0.001†
Baseline MDS-UPDRS-III - Rigidity	3.61 (2.28)	5.17 (2.96)	0.057†	3.87 (2.40)	5.67 (3.31)	0.107†
Baseline MDS-UPDRS-III - Posture & Gait	0.42 (0.50)	1.56 (1.15)	<0.001†	0.39 (0.50)	1.83 (1.19)	<0.001†
Baseline MoCA	27.29 (1.82)	27.17 (2.68)	0.784†	27.30 (1.89)	26.67 (3.09)	0.453
Testing/MRI Sites	9	10	n/a	8	8	n/a
FW Caudate	0.248 (0.031)	0.260 (0.039)	0.279	0.245 (0.032)	0.261 (0.040)	0.211†
FW Putamen	0.160 (0.030)	0.162 (0.043)	0.685†	0.159 (0.030)	0.160 (0.034)	0.945†
FW GP	0.165 (0.060)	0.157 (0.040)	0.928†	0.173 (0.063)	0.147 (0.029)	0.248†
FW STN	0.109 (0.025)	0.103 (0.025)	0.404	0.115 (0.025)	0.100 (0.028)	0.114
FW ASN	0.198 (0.054)	0.192 (0.077)	0.514†	0.206 (0.056)	0.199 (0.093)	0.366†
FW PSN	0.151 (0.039)	0.144 (0.045)	0.392†	0.151 (0.040)	0.148 (0.050)	0.602†
FW Thalamus	0.122 (0.020)	0.116 (0.018)	0.331	0.125 (0.020)	0.121 (0.017)	0.637
FW PPN	0.108 (0.032)	0.110 (0.031)	0.825	0.110 (0.032)	0.111 (0.037)	0.909
FW RN	0.104 (0.012)	0.097 (0.019)	0.062†	0.106 (0.012)	0.095 (0.017)	0.050
FW Lobule V	0.167 (0.021)	0.168 (0.023)	0.842	0.169 (0.020)	0.169 (0.027)	0.947
FW Lobule VI	0.152 (0.019)	0.154 (0.023)	0.764	0.152 (0.019)	0.154 (0.025)	0.760
FW Vermis	0.200 (0.027)	0.208 (0.023)	0.260	0.201 (0.028)	0.214 (0.022)	0.181
FW Dentate Nucleus	0.160 (0.028)	0.155 (0.028)	0.540	0.163 (0.029)	0.151 (0.029)	0.258

Variables except for sample size and sex are presented as mean (±SD). MDS-UPDRS-III, motor section of the Movement Disorders Society Unified Parkinson’s Disease Rating Scale; MoCA, Montreal Cognitive Assessment Test; MRI, magnetic resonance imaging; *N*, number; PD, Parkinson’s disease; PIGD, posture instability and gait difficulties; TD, tremor dominant; *Y*, years. Value of *p*s correspond to the statistical tests used to compare baseline characteristics between TD and PIGD. A dagger symbol indicates the results of non-parametric between-group comparisons. An asterisk indicates that group differences were significant at a *p* < 0.05.

### 2.2. Diffusion MRI data acquisition

The diffusion MRI data were acquired at multiple sites (Table 1) on 3 T Tim Trio Siemens scanners equipped with a 12-channel matrix head coil and running software versions VB15. The diffusion tensor imaging (DTI) sequence used in the PPMI 1.0 protocol was had the following parameters: TR = 900 ms (cardiac-gated, according to individual heart rate), TE = 88 ms, matrix = 116 × 116, flip angle = 90°, slice order = interleaved, 64 gradient directions with a *b*-value of 1,000 s/mm^2^, one b = 0 s/mm^2^, and a voxel size of 1.98 × 1.98 × 2.0 mm.

### 2.3. Diffusion MRI data analysis

#### 2.3.1. Preprocessing

Data preprocessing followed established protocols used in prior PD studies where FW was employed as the primary outcome measure (21, 27, 28, 30). To preprocess the data, we employed the FMRIB Software Library (FSL; Oxford, United Kingdom) and custom UNIX shell scripts. The preprocessing pipeline involved removing non-brain tissue, correcting the signal in the DTI data for eddy-current and head motion-induced distortions, and rotating the gradient vectors based in response to these corrections. Next, FW maps were calculated from the preprocessed data using a custom code written in MATLAB (The Mathworks, Natick, MA). During this calculation, a bi-tensor model was fit to each voxel instead of a single-tensor model to quantify the fractional volume of FW for that voxel (23). Within a voxel, there can be a mixture of different tissue types, such as brain tissue and cerebrospinal fluid. Partial volume effects occur when a voxel contains a mix of these tissue types. The bi-tensor model distinguishes the diffusion characteristics of water in brain tissues from those in the extracellular space. This differentiation is significant as it prevents the extracellular space from influencing the diffusion values obtained through the single-tensor model. The isolated FW contains vital structural information, which is useful for studying neurodegenerative diseases. The *b*-zero image of each participant was normalized to a DTI template used in recent PD studies (30, 38, 39) using a non-linear registration approach (SyN) available in the Advanced Normalization Tools package (ANTs) (40) The resulting transformation matrix was then applied to the FW maps. The final FW maps in MNI space had a resolution of 2 mm isotropic.

#### 2.3.2. Regions of interest

**Figure 1:**
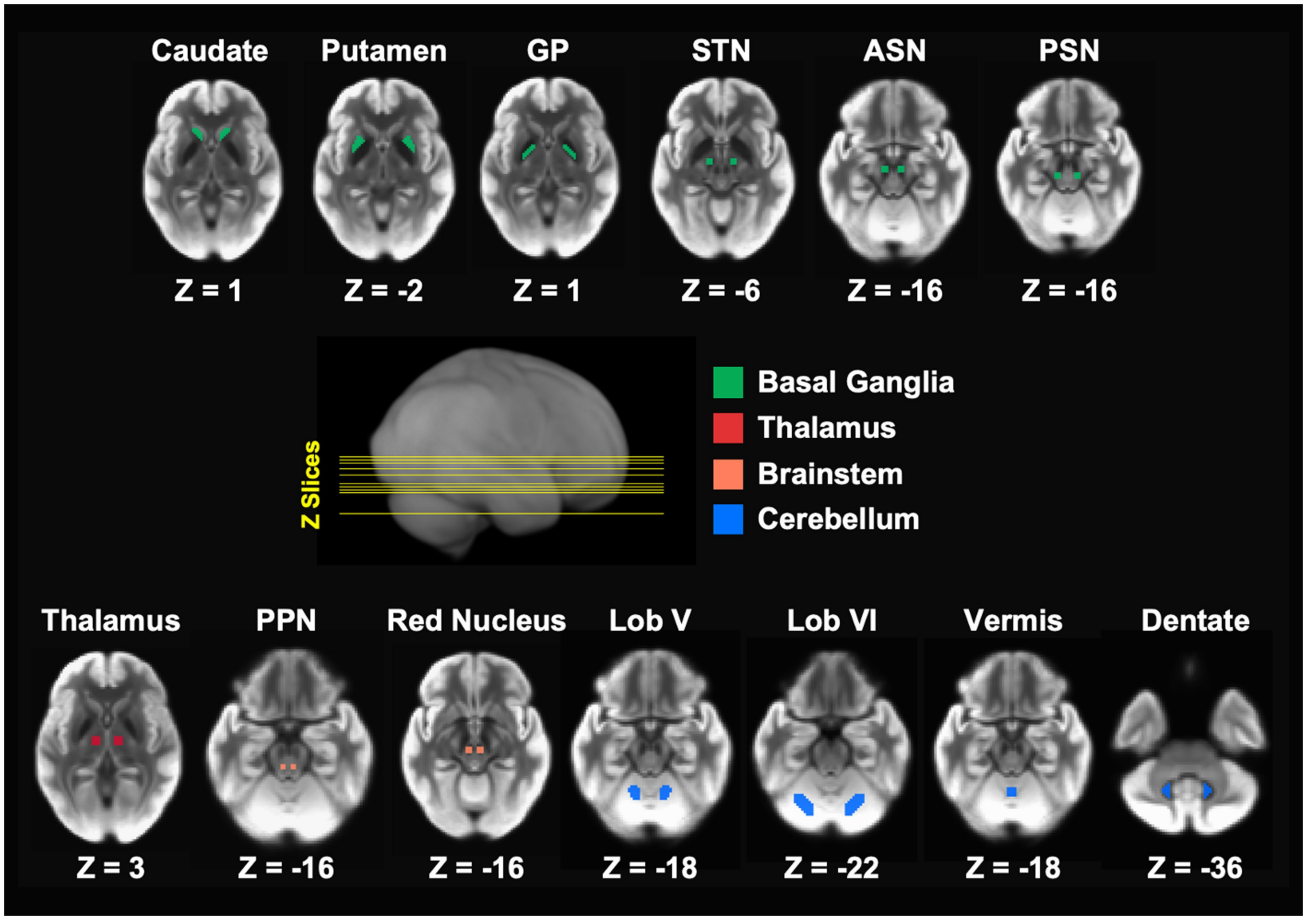
Bilateral regions of interest (ROIs) were used in the FW analysis. The ROIs spanned the basal ganglia, thalamus, brainstem, and cerebellar, and are in MNI space. ASN, anterior substantia nigra; GP, globus pallidus; Lob V, cerebellar lobule V; Lob VI, cerebellar lobule VI; MNI, Montreal Neurological Institute; PPN, pedunculopontine nucleus; PSN, posterior substantia nigra; STN, subthalamic nucleus.

All Regions of interest (ROIs) for the DTI analysis were in MNI space and placed bilaterally in brain structures involved in motor control across the basal ganglia, thalamus, brainstem, and cerebellum (Figure 1). ROIs in the basal ganglia included the caudate nucleus, putamen, GP, STN, anterior substantia nigra (ASN), and PSN. Additional ROIs were placed in the thalamus, pedunculopontine nucleus (PPN), red nucleus (RN), and several motor regions within the cerebellum including lobule V, lobule VI, anterior vermis, and the dentate nucleus.

All ROIs except for ASN, PSN, and vermis were obtained from a validated template of ROIs implicated in PD (30). In a previous study that investigated the 4 years progression of FW in ASN and PSN in PD from the PPMI study, (27) we employed slightly smaller ROIs than those included in the aforementioned template. These ROIs, each spanning two slices and comprising 16 voxels (voxel size: 2 × 2 × 2 mm), were manually drawn for each participant at each timepoint on the normalized *b*-zero scan by two experienced raters who were blinded to group status, timepoint, and the FW values. Considering the improved within- and between-subject alignment of brain structures, including smaller regions like the substantia nigra, achieved through the ANTs normalization, we created one set of ASN and PSN ROIs in MNI space using the anatomical landmarks described in our previous work (27). This approach allowed for a more appropriate comparison of the current PPMI data with the PPMI data used in our previous study (27). Finally, in the template mentioned above, the vermis ROI comprised the posterior vermis in the posterior lobe of the cerebellum. Here, we are using an ROI that consists of the anterior vermis in the anterior lobe of the cerebellum. It is well-known that the anterior cerebellar vermis plays a crucial role in coordinating gait and facilitating balance and postural adjustment of the trunk and legs (41, 42). Such motor behaviors are affected in the PIGD subtype (43, 44). ROIs were used to extract FW values for each participant at baseline and year 2.

### 2.4. Statistical analysis

Statistical analyses for both clinical and imaging data were performed using SPSS 28.0 (IBM, New York). Outcome measures included clinical and FW measures at baseline and the change in these measures over the two-year period. Since using the absolute difference in values may not account for individual baseline variations and could lead to confounding effects and difficulties in the interpretation of the data, we decided to conduct statistical analysis on the % change. Percent change was calculated by subtracting the baseline value from the year two value, dividing the new value by the baseline value, and multiplying it by 100. This was done for FW values in all ROIs for Cohorts 1 and 2, as well as for the total MDS-UPDRS-III score for Cohort 2.

First, the normality and equal variance were assessed for all data using Shapiro–Wilk and Levene’s tests. The results of these tests helped determine whether further analyses would utilize parametric or non-parametric testing. Categorical data such as sex (Table 1) were compared between the TD and PIGD groups using the Chi-Square test. Group differences in the remaining continuous variables at baseline (clinical measures and FW values in all ROIs) were assessed with Independent Samples *t*-tests or Mann–Whitney U Tests. For Cohort 2, group differences in the % change in total MDS-UPDRS-III were assessed with an Independent Samples *t*-test. Differences between TD and PIGD in the progression of FW in all ROIs were assessed using an Analysis of Covariance (ANCOVA) with the testing (MRI) site as a covariate for Cohort 1, and testing site and % change in total MDS-UPDRS-III for Cohort 2. In the latter analysis, the % change in total MDS-UPDRS-III was used as a covariate because previous studies have suggested that individuals with the PIGD subtype may exhibit a faster progression of motor symptoms compared to those with a TD subtype (16). By using the change in clinical scores as a covariate, we aimed to control for the potential confounding effect of motor symptom progression on group differences in brain measures between TD and PIGD subtypes.

Finally, we conducted partial correlation analyses to investigate the potential relationship between clinical changes (i.e., changes in the severity of motor symptoms) and FW changes in any brain regions where the progression of FW differed between TD and PIGD subtypes. The partial correlations controlled for the testing site (data in Cohort 2). Three logistic regression analyses were also conducted on data from Cohort 2 to predict the probability of a PD belonging to one of the two PD subtypes. These analyses used different sets of features: change in clinical measures only (i.e., % change in total MDS-UPDRS-III), changes in FW only, and changes in clinical and FW. Results were noted as significant if value of *p*s < 0.05.

## 3. Results

Below are the results of several statistical analyses focused on assessing: (1) differences between TD and PIGD in clinical and FW measures at baseline, (2) differences between TD and PIGD in the progression over 2 years of motor symptoms, (3) differences between TD and PIGD in the progression over 2 years of FW, (4) the relationship between the progression over 2 years of motor symptoms and that of FW, evaluated separately in TD and PIGD, and (5) whether longitudinal changes in motor symptoms, FW, or a combination of both are able to predict PD subtype.

### 3.1. Baseline comparisons

Baseline comparisons of the TD and PIGD groups within each cohort revealed no significant differences between groups in age, sex, severity of motor symptoms, or cognitive status (*p* values > 0.050; Table 1). Similarly, within each cohort, the baseline FW values in all ROIs did not show any significant differences between the TD and PIGD groups: caudate nucleus (Cohort 1 *p*_(T-Test)_ = 0.279; Cohort 2 *p*_(Mann–Whitney)_ = 0.211), putamen (Cohort 1 *p*_(Mann–Whitney)_ = 0.685; Cohort 2 *p*_(Mann–Whitney)_ = 0.945), GP (Cohort 1 *p*_(Mann–Whitney)_ = 0.928; Cohort 2 *p*_(Mann–Whitney)_ = 0.248), STN (Cohort 1 *p*_(T-Test)_ = 0.404; Cohort 2 *p*_(T-Test)_ = 0.114), ASN (Cohort 1 *p*_(Mann–Whitney)_ = 0.514; Cohort 2 *p*_(Mann–Whitney)_ = 0.366), PSN (Cohort 1 *p*_(Mann–Whitney)_ = 0.392; Cohort 2 *p*_(Mann–Whitney)_ = 0.602), thalamus (Cohort 1 *p*_(T-Test)_ = 0.331; Cohort 2 *p*_(T-Test)_ = 0.637), PPN, (Cohort 1 *p*_(T-Test)_ = 0.825; Cohort 2 *p*_(T-Test)_ = 0.909), RN (Cohort 1 *p*_(Mann–Whitney)_ = 0.062; Cohort 2 *p*_(T-Test)_ = 0.050), cerebellar lobule V (Cohort 1 *p*_(T-Test)_ = 0.842; Cohort 2 *p*_(T-Test)_ = 0.947), cerebellar lobule VI (Cohort 1 *p*_(T-Test)_ = 0.764; Cohort 2 *p*_(T-Test)_ = 0.760), cerebellar vermis (Cohort 1 *p*_(T-Test)_ = 0260; Cohort 2 *p*_(T-Test)_ = 0.181), and cerebellar dentate nucleus (Cohort 1 *p*_(T-Test)_ = 0.540; Cohort 2 *p*_(T-Test)_ = 0.258) (Table 1).

### 3.2. Progression of motor symptoms

An independent samples *t*-test comparing the percent change in total MDS-UPDRS-III off medication over the 2 years period between TD and PIGD revealed a greater increase in the severity of motor symptoms in PIGD compared to TD (*p*_(T-Test)_ = 0.004; total MDS-UPDRS-III TD_Baseline_ = 21.82 (±8.09), total MDS-UPDRS-III TD_Year2_ = 24.04 (±10.27); total MDS-UPDRS-III PIGD_Baseline_ = 25.33 (±9.54); total MDS-UPDRS-III PIGD_Year2_ = 38.25 (±14.49)). Of note, this analysis was conducted on data from Cohort 2 as not all PD in Cohort 1 had a total MDS-UPDRS-III off medication at year 2 (Table 2).

**Table 2:** FW measures were extracted from each ROI at baseline and 2 years later for Cohorts 1 and 2.

SCORE/ROI	TD			PIGD		
	Baseline	Year 2	% Change	Baseline	Year 2	% Change
MOTOR SEVERITY						
Total MDS-UPDRS-III	21.82 (8.09)	24.04 (10.27)	12.48 (32.27)	25.33 (9.54)	38.25 (14.49)	59.28 (57.39)
FREE WATER						
COHORT 1						
FW Caudate	0.248 (0.031)	0.251 (0.028)	1.37 (6.34)	0.260 (0.039)	0.267 (0.033)	3.15 (7.11)
FW Putamen	0.160 (0.030)	0.159 (0.029)	0.15 (9.66)	0.162 (0.043)	0.193 (0.076)	17.19 (19.76)
FW GP	0.165 (0.060)	0.164 (0.054)	1.56 (13.78)	0.157 (0.040)	0.176 (0.053)	12.08 (16.06)
FW STN	0.109 (0.025)	0.115 (0.030)	7.57 (27.41)	0.103 (0.025)	0.110 (0.031)	8.89 (23.98)
FW ASN	0.198 (0.054)	0.192 (0.035)	1.55 (24.34)	0.192 (0.077)	0.188 (0.053)	5.12 (33.34)
FW PSN	0.151 (0.039)	0.155 (0.044)	4.39 (21.40)	0.144 (0.045)	0.151 (0.044)	7.23 (25.71)
FW Thalamus	0.122 (0.020)	0.122 (0.021)	1.72 (18.21)	0.116 (0.018)	0.130 (0.029)	12.72 (21.75)
FW PPN	0.108 (0.032)	0.112 (0.042)	10.90 (53.33)	0.110 (0.031)	0.114 (0.031)	7.16 (24.13)
FW RN	0.104 (0.012)	0.107 (0.019)	4.10 (16.75)	0.097 (0.019)	0.105 (0.015)	10.13 (18.09)
FW Lobule V	0.167 (0.021)	0.168 (0.023)	1.32 (13.36)	0.168 (0.023)	0.172 (0.027)	3.93 (10.41)
FW Lobule VI	0.152 (0.019)	0.155 (0.024)	1.96 (8.76)	0.154 (0.023)	0.157 (0.025)	2.31 (9.99)
FW Vermis	0.200 (0.027)	0.201 (0.026)	1.49 (12.30)	0.208 (0.023)	0.212 (0.031)	1.89 (12.38)
FW Dentate Nucleus	0.160 (0.028)	0.154 (0.025)	−1.93 (18.93)	0.155 (0.028)	0.158 (0.031)	4.29 (23.87)
COHORT 2						
FW Caudate	0.245 (0.032)	0.246 (0.027)	0.841 (6.09)	0.261 (0.040)	0.267 (0.028)	3.38 (8.09)
FW Putamen	0.159 (0.030)	0.158 (0.026)	−0.16 (8.73)	0.160 (0.034)	0.183 (0.053)	14.50 (21.46)
FW GP	0.173 (0.063)	0.171 (0.057)	0.039 (11.36)	0.147 (0.029)	0.161 (0.037)	10.09 (18.23)
FW STN	0.115 (0.025)	0.118 (0.032)	5.72 (29.23)	0.100 (0.028)	0.108 (0.034)	10.16 (26.82)
FW ASN	0.206 (0.056)	0.195 (0.037)	−0.79 (25.55)	0.199 (0.093)	0.190 (0.049)	6.10 (35.83)
FW PSN	0.151 (0.040)	0.151 (0.040)	3.00 (20.32)	0.148 (0.050)	0.146 (0.041)	2.08 (25.23)
FW Thalamus	0.125 (0.020)	0.124 (0.022)	0.38 (17.10)	0.121 (0.017)	0.132 (0.030)	9.65 (24.37)
FW PPN	0.110 (0.032)	0.108 (0.039)	2.08 (33.49)	0.111 (0.037)	0.119 (0.032)	11.25 (24.87)
FW RN	0.106 (0.012)	0.110 (0.019)	4.38 (17.16)	0.095 (0.017)	0.108 (0.014)	15.12 (19.17)
FW Lobule V	0.169 (0.020)	0.168 (0.024)	−0.43 (11.38)	0.169 (0.027)	0.175 (0.030)	4.32 (11.37)
FW Lobule VI	0.152 (0.019)	0.153 (0.023)	0.95 (7.07)	0.154 (0.025)	0.158 (0.024)	2.83 (9.13)
FW Vermis	0.201 (0.028)	0.202 (0.027)	1.17 (12.02)	0.214 (0.022)	0.211 (0.035)	−1.05 (11.84)
FW Dentate Nucleus	0.163 (0.029)	0.156 (0.023)	−2.15 (19.39)	0.151 (0.029)	0.154 (0.030)	4.59 (26.89)

Variables are presented as mean total MDS-UPDRS-III/FW and % change in the same variables (±SD). ASN, anterior substantia nigra; GP, globus pallidus; PIGD, posture instability and gait difficulties; PPN, pedunculopontine nucleus; PSN, posterior substantia nigra; ROI, region of interest; RN, red nucleus; STN, subthalamic nucleus; TD, tremor dominant.

### 3.3. Progression of FW

**Figure 2:**
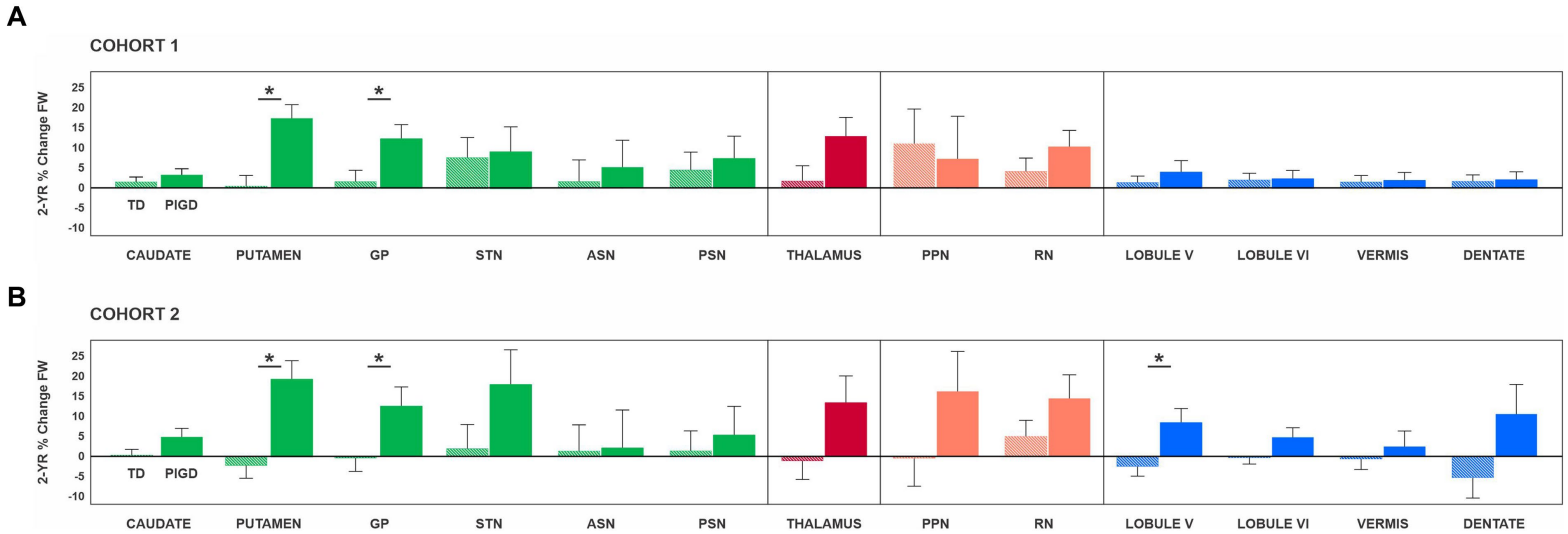
Results of the analysis of covariance are presented separately for Cohort 1 **(A)** and Cohort 2 **(B)**. Of note, Cohort 1 included PD who had DTI scans at baseline and 2 years later, while Cohort 2 included PD who had DTI scans and the total MDS-UPDRS-III score off medication at baseline and 2 years later. For Cohort 1, the analyses included the MRI site as a covariate, while for Cohort 2, the covariates were the MRI site and % change in total MDS-UDPRS-III. Asterisks indicate statistically significant differences between TD and PIGD in % change FW. Error bars represent ±1 standard error. ASN, anterior substantia nigra; GP, globus pallidus; MDS-UPDRS-III, motor section of the Movement Disorders Society Unified Parkinson’s Disease Rating Scale; PIGD, posture instability and gait difficulties; PPN, pedunculopontine nucleus; PSN, posterior substantia nigra; RN, red nucleus; STN, subthalamic nucleus; TD, tremor dominant.

An ANCOVA was conducted separately for each ROI to determine whether there were any statistically significant differences between TD and PIGD in the % change in FW over the two-year period. For Cohort 1, the analyses included testing site as a covariate, while for Cohort 2, the covariates were testing site and % change in total MDS-UDPRS-III. The FW values at baseline and year 2 for each ROI can be found in Table 2, while the results of the ANCOVA analyses for each ROI are presented in Figure 2.

For Cohort 1, a significant effect of PD subtype on % change in FW was found for the putamen (*F*_(1,43)_ = 15.01; *p* < 0.001; *η*_p_^2^ = 0.259) and GP (*F*_(1,43)_ = 5.47; *p* = 0.024; *η*_p_^2^ = 0.113) (Figure 2A). Specifically, the PIGD group had a greater % change in FW than the TD group in the putamen (PIGD = 17.19 ± 3.40%; TD = 0.15 ± 2.72%) and GP (PIGD = 12.07 ± 3.46%; TD = 1.56 ± 2.77%). For both ROIs, there was no significant testing site effect (*p* values >0.05), indicating that the MRI scanner did not have an impact on the FW measures. There were no significant differences in the progression of FW over 2 years between TD and PIGD in the remaining ROIs: caudate nucleus (*p* = 0.381), STN (*p* = 0.858), ASN (*p* = 0.684), PSN (*p* = 0.692), thalamus (*p* = 0.074), PPN, (*p* = 0.781), RN (*p* = 0.261), cerebellar lobule V (*p* = 0.490), cerebellar lobule VI (*p* = 0.917), cerebellar vermis (*p* = 0.923), and cerebellar dentate nucleus (*p* = 0.338) (Figure 2A).

In Cohort 2, when accounting for the progression of motor symptoms, we identified significant differences between the two subtypes in the putamen (*F*(_1,31)_ = 12.81; *p* < 0.001; *η*_p_^2^ = 0.293), GP (*F*(_1,31)_ = 4.28; *p* = 0.047; *η*_p_^2^ = 0.121), and cerebellar lobule V (*F*(_1,31)_ = 5.46; *p* = 0.026; *η*_p_^2^ = 0.150) (Figure 2B). Specifically, the PIGD group had a greater % change in FW than the TD group in the putamen (PIGD = 14.50 ± 21.45%; TD = −0.16 ± 8.73%), GP (PIGD = 10.09 ± 18.23%; TD = 0.38 ± 11.36%), and cerebellum (PIGD = 4.32 ± 11.36%; TD = −0.42 ± 11.38%). Consistent with the findings from Cohort 1, the testing site effect was not statistically significant (*p* values >0.05). No statistically significant differences were found in the progression of FW over 2 years between TD and PIGD in the other ROIs: caudate nucleus (*p* = 0.102), STN (*p* = 0.165), ASN (*p* = 0.950), PSN (*p* = 0.676), thalamus (*p* = 0.105), PPN, (*p* = 0.212), RN (*p* = 0.221), cerebellar lobule VI (*p* = 0.141), cerebellar vermis (*p* = 0.594), and cerebellar dentate nucleus (*p* = 0.108) (Figure 2B).

In a previous longitudinal analysis, which focused on assessing FW changes in a larger group of PD participants from the PPMI study over a 4 years follow-up period, FW was found to be significantly elevated in the PSN at follow-up (year 1, 2, and 4) compared to baseline. In this study, although there was an increase in FW values in the PSN, particularly more prominent in the PIGD group (Figure 2), this difference was not statistically significant. To gain further insights into the progression of FW in the PSN of both TD and PIGD subtypes, we conducted an additional partial correlation analysis. This analysis explored the possible relationship between baseline FW in the PSN and the % change in FW in the PSN across both groups while controlling for testing site and the potential confounding effect of change in motor symptoms (i.e., % change in total MDS-UPDRS-III). This correlation demonstrated that across groups baseline FW values in the PSN were negatively correlated to % change in FW in the PSN over 2 years (rho = −0.573, *p* < 0.001). That is, PD who had a higher FW in the PSN at baseline experienced a lower % change in FW in the same area over 2 years.

### 3.4. Relationship between the progression of motor symptoms and the progression of FW changes. Predicting PD subtype

The % change in the total MDS-UPDRS-III score over 2 years did not correlate with the percent change in FW in the putamen, GP, or cerebellar lobule V in TD or PIGD (*p* values >0.05).

A first logistic regression model using only the % change in total MDS-UPDRS-III as a predictor of PD subtype revealed a moderate ability of the rate of progression of motor symptoms to discriminate between TD and PIGD subtypes. The model was statistically significant (*X*^2^ = 8.85, *p* = 0.003), explained 31.2% of the variance (Nagelkerke *R*^2^), and correctly classified 74.3% of cases (91.3% TD vs. 41.7% PIGD). The second logistic regression model using only the % change in FW in the putamen, GP, and cerebellar lobule V, had a slightly better ability to discriminate TD from PIGD: *X*^2^ = 15.34, *p* = 0.002, Nagelkerke *R*^2^ = 0.384, and correctly classified 76.1% of cases (89.3% TD vs. 55.6% PIGD). Finally, the third model which tested the predictive ability of the rate of progression of both clinical measures and structural brain changes (i.e., % change in total MDS-UPDRS-III and % change in FW in the putamen/GP/cerebellar lobule V), demonstrated superior discrimination power to distinguish between PD subtypes compared to the models based solely on clinical measures or imaging measures (*X*^2^ = 26.48, *p* = <0.001, Nagelkerke *R*^2^ = 0.734, and correctly classified 91.4% of cases – 95.7% TD vs. 83.3% PIGD).

## 4. Discussion

In this study, we conducted the first investigation of the progression of FW over a span of 2 years across multiple motor-related brain regions in the TD and PIGD subtypes. The study revealed several novel findings. First, we confirmed a more rapid progression of motor symptoms in PIGD compared to TD, within a cohort of early-stage PD from the PPMI study who were untreated at enrollment and had a clinical diagnosis confirmed by DaTscan. Second, PIGD had a faster rate of increase in FW within regions of the basal ganglia and cerebellum when contrasted with TD, a finding which was consistent across MRI sites. Third, although changes in PSN showed no significant between-group differences and were smaller in PIGD compared to changes in other basal ganglia regions, they were related to the initial FW value. In other words, higher FW values in PSN at baseline were associated with a smaller change over time. Finally, a logistic regression model demonstrated that the % change in FW within the putamen, GP, and cerebellum lobule V, coupled with the % change in the total MDS-UPRDS-III score off medication, could distinguish PD subtypes with increased accuracy (91.4%). This model’s discrimination power surpassed that of two other models incorporating solely clinical measures or imaging measures.

### 4.1. Progression of motor symptoms in TD vs. PIGD

The results of this study build upon previous research (9, 16) and demonstrate that individuals with a PIGD subtype have a faster progression of motor symptoms (here, assessed off medication) over a 2 years period compared to those with a TD subtype. One notable strength of this analysis is that these observations are in PD untreated at enrollment and are not confounded by baseline measures. That is, both groups started off with similar clinical scores and structural brain measures. In terms of the long-term clinical outcomes, there has been speculation that individuals with PIGD may experience a broader range of pathophysiological changes extending beyond the basal ganglia than TD. These changes could potentially contribute to the observed faster deterioration in motor function in this subgroup. To the best of our knowledge, our study is the first to investigate longitudinal microstructural changes in PD subtypes, with a focus on multiple brain regions involved in motor control: basal ganglia, thalamus, brainstem, and cerebellum.

### 4.2. Progression of structural brain changes in TD vs. PIGD

The analysis of FW data from Cohort 1 revealed a greater rate of FW increase over a 2 years period in PIGD compared to TD in the putamen and globus pallidus. It is worth noting that there were no initial differences in FW between the two groups within any of the ROIs. The putamen serves as an input structure to the basal ganglia and its activity is shaped by the release of dopamine from the substantia nigra pars compacta (5, 45), Conversely, GP assumes the role of the output structure of the basal ganglia, exercising influence over the thalamus (10, 45, 46). Both structures are an integral part of the motor circuit and are involved in the pathophysiology of PD (47–49). Recent work based on the PPMI data identified distinct patterns in the progression of dopaminergic denervation based on striatal binding ratios (SBR) derived from I-123 FP-CIT SPECT images in the two PD subtypes. Specifically, the putaminal SBR of PIGD remained consistently lower than that of TD for the first 2 years of the follow-up (50). As for structural differences, previous cross-sectional volumetric and DTI studies revealed greater gray matter loss and increased diffusivity in GP in PIGD compared to TD (51). Importantly, the more severe the symptoms in PIGD, the higher these diffusivity levels, (51) suggesting that structural changes within GP worsen with disease progression in the PIGD subtype. Here, we extend the literature by showing a significant progression-related decline in the microstructural integrity of several basal ganglia regions in PIGD but not in TD (Figure 2).

Because TD and PIGD in this study experience distinct rates of motor symptom progression over the 2 year period, we conducted an additional analysis in Cohort 2 (PD who had both imaging and clinical scores off medication) to be able to relate changes in brain structure to changes in clinical scores, and most importantly, to isolate the true effects of PD subtype on brain changes. By including the rate of symptom progression as a covariate in our analysis, we wanted to ensure that any observed group differences in brain changes are not confounded by differences in disease severity. In this complementary analysis, we confirm that PIGD have a greater % change in FW compared to TD in the putamen, GP, and cerebellum. An increasing amount of literature is pointing to the potential involvement of the cerebellum in PD (52–54). It remains to be fully understood whether changes in the cerebellum are part of the pathophysiology of the disease or if the cerebellum functions as a compensatory mechanism in response to nigral degeneration. Lobule V of the cerebellum is a key component of the motor cerebellum which is interconnected with cortical brain regions involved in motor planning, execution, and coordination (53, 55, 56). In previous work, cerebellar atrophy was found in PD tested about 5 years post-diagnosis, and this finding was accompanied by abnormalities in cerebellar-cortical functional connectivity (57). While the subtype of these PD is not known, it could be that the observed effects may be driven by or be more prominent in PD with a PIGD subtype who exhibit abnormalities in balance control, walking, and motor coordination, all functions regulated by the cerebellum. Here, FW may be able to capture additional information about the microstructural integrity of the cerebellum and produce a more detailed picture of cerebellar changes in PD. Given recent evidence that neuroinflammation precedes neurodegeneration in PD and that increased FW relates to higher TSPO-PET markers of neuroinflammation (58), it could be that FW changes within the cerebellum occur prior to evident atrophy within this region. It is important to note a trend for PIGD to exhibit a progression of FW levels not only in the putamen, GP, and cerebellar lobule V but also in other structures of the basal ganglia, cerebellum, as well as thalamus, and brainstem. Although these changes did not reach statistical significance, they seem to point to PIGD being a multi-system disorder affecting various levels of the motor network. These widespread patterns of changes may explain the accelerated progression of symptoms. The faster deterioration of PIGD patients may also be attributed to the fact that a significant proportion of these brain regions do not fall within the category of dopaminergic sites that one would expect to respond to conventional PD medication. Interestingly, a study conducted on participants from the PPMI study followed up for 1 year, demonstrated that dopaminergic therapy does not impact the allocation to one PD subtype (59). This finding further supports the hypothesis that motor symptoms in PIGD might be rooted in brain changes extending beyond the basal ganglia.

As for changes within the substantia nigra, we found no significant differences between groups in the 2 years % change increase in FW in PSN or ASN. Nevertheless, as illustrated in Figure 2, there is a tendency for the increase in FW to be more pronounced in the PIGD group. Previous single-and multi-site studies of PD progression have repeatedly demonstrated an increase in FW in the PSN at the 1 year, 2 years, and 4 years marks in early-stage PD (26, 27). However, in a study tracking the progression of FW in PD with a mean disease duration of ca. 7 years, only the ASN FW showed an increase and not the PSN, suggesting that changes in FW in PSN may plateau after several years (60). Indeed, it seems that individuals with PD undergo a substantial decline in dopamine-producing cells within the first four years of the disease, after which there is little change in the count of dopaminergic cells (2). An interesting finding in our study was the correlation across groups between baseline FW and the 2 years % change in FW in PSN. This revealed that those PD who had higher FW in PSN at baseline had a slower progression in FW levels compared to those PD who started off with a lower value. One potential explanation for the elevated FW levels at baseline in some individuals is the heterogeneity in the progression rates of the neurodegenerative process which is known to start years before the first motor symptoms emerge. In this context, measures such as disease duration should be interpreted with caution. Although the baseline scan was conducted close to the date of the diagnosis, it is possible that some PD were way ahead in the disease course. Disease duration in studies such as the PPMI reflects the time since diagnosis rather than the time since symptom onset. The latter is difficult to estimate since many patients delay seeking medical attention for their symptoms. Taken together, these findings suggest that FW could serve as a valuable metric for investigating progression-related changes in the brain of PD subtypes. However, its sensitivity to detecting longitudinal changes in TD and PIGD requires confirmation in future, larger-scale studies.

An important research question we wanted to address in this study was whether there was any connection between changes in motor scores and changes in FW levels. The lack of a correlation between the two was not unexpected. Rather, it implies that the extensive structural changes observed in PIGD could potentially give rise to additional motor symptoms that are not encompassed by MDS-UPDRS-III. Moreover, it is plausible that multiple contributing factors such as genetic makeup may predispose individuals to more accelerated brain deterioration. Also, it is important to acknowledge the MDS-UPDRS-III emphasizes symptoms related to the upper limbs and does not comprehensively address symptoms affecting other body parts, such as balance and gait disturbances that are more pronounced in the PIGD subtype. Interestingly, a logistic regression model based solely on the % change in motor severity exhibited limited discriminatory power, highlighting the shortcomings of the clinical scale. By contrast, a superior classification of the two subtypes was achieved by combining % change in motor severity with % changes in FW in the putamen, GP, and cerebellar lobule V. This model correctly classified 91.4% of the cases, with a breakdown of 95.7% for TD and 83.3% for PIGD. The model incorporating solely the imaging measures demonstrated a moderate performance. However, more work needs to be conducted in this area of classification models and results need to be confirmed in larger cohorts. Alternative classification models could include decision trees and random forest models that rely on training and testing datasets.

Finally, this study is not without limitations. It has been previously acknowledged that there is a certain degree of variability in the stability of PD subtypes in *de novo* PD, especially during the first year since diagnosis (about 61% of PIGD and 82% of TD remained in their respective subtypes after 1 year) (59, 61). Here, we implemented more rigorous inclusion criteria to counteract this phenomenon. We extended the follow-up period to 2 years and only included PD patients who exhibited the same subtype at both time points. Moreover, we verified the stability of these PD subtypes beyond the 2 years mark in those PD who had total MDS-UPDRS-III scores off medication at year 4 (majority of PD). It is important to mention, that despite our initial intention to do so, we were unable to perform an analysis that would assess brain changes over a 4 years period. The number of PIGD patients with a consistent subtype for a longer period in the PPMI dataset is relatively small as noted in another study (9), and the number of PIGD who have both clinical scores off medication and imaging data is even smaller. Another common challenge in longitudinal studies focused on PD is that of managing variables such as the patient’s medication regimens and the degree of physical activity. Frequently, the information pertaining to the medication is either incomplete or absent, the time when individuals are started on medication varies, and data regarding their level of physical activity is unavailable. Although no study to date indicates a positive effect of these variables (i.e., drugs or exercise) on FW levels (disease-modifying drugs are yet to be identified), it cannot be ruled out that the absence of/limited changes in FW in TD (Figure 2) may be linked to a more favorable response of tremor symptoms to PD medication.

Future studies examining the differential disease course of the TD and PIGD subtypes should consider: (1) extending the follow-up, (2) documenting with greater detail the medication taken during the follow-up period and the type and amount of exercise PD engage in, (3) incorporating multi-modal imaging, (4) incorporating conventional DTI measures such as fractional anisotropy (FA) to assess the sensitivity of different DTI-based metrics in detecting longitudinal microstructural brain changes across PD subtypes, (5) focusing the analyses on PD subtypes experiencing more advanced stages of the disease, and (6) expanding the analyses to include ROIs beyond the motor circuit, as non-motor symptoms tend to become more prevalent with disease progression, particularly in the PIGD subtype. Finally, considering subtypes when designing clinical trials or factoring in subtypes in the analysis of the clinical trial data may prove to be a valuable strategy that could limit the potential confounding effects of PD subtypes on outcome measures.

## 5. Conclusion

The primary goal of this study was to compare the rate of FW increase over a 2 years period in multiple motor regions across the basal ganglia, thalamus, brainstem, and cerebellum between TD and PIGD. We found that when compared to TD, PIGD experienced a faster clinical deterioration and changes in FW that spanned both the basal ganglia and cerebellum. In conjunction with changes in clinical scores, these measures distinguished the two subtypes with increased accuracy, superior to that of models based solely on clinical or imaging measures. In summary, the outcomes of this study serve to reinforce the validity and utility of FW as a marker of disease progression in PD and offer valuable insights into the distinct trajectories of disease progression within PD subtypes. The latter has implications for the design of pharmaceutical and non-pharmaceutical intervention studies aimed at addressing the specific needs of these PD subtypes.

## Data availability statement

The data analyzed in this study was obtained from the Parkinson’s Progressive Marker Initiative (PPMI; http://www.ppmi-info.org/), the following licenses/restrictions apply: Investigators seeking access to PPMI data must sign the Data Use Agreement, submit an Online Application and comply with the study Publications Policy. Requests to access these datasets should be directed to PPMI, https://ida.loni.usc.edu/collaboration/access/appLicense.jsp.

## Ethics statement

Data included in this study was obtained from the Parkinson’s Progressive Marker Initiative (PPMI; http://www.ppmi-info.org/) study. The IRB of each participating site approved this study. The studies were conducted in accordance with the local legislation and institutional requirements. The participants provided their written informed consent to participate in this study.

## Author contributions

AB: Formal analysis, Methodology, Writing – original draft, Writing – review & editing. SC: Formal analysis, Methodology, Writing – original draft, Writing – review & editing. JC: Writing – original draft, Writing – review & editing. JM: Writing – original draft, Writing – review & editing. RB: Conceptualization, Formal analysis, Methodology, Supervision, Writing – original draft, Writing – review & editing.

## References

[1] Poewe W Seppi K Tanner CM Halliday GM Brundin P Volkmann J. Parkinson disease. Nat Rev Dis Primer. (2017) 3:17013. doi: 10.1038/nrdp.2017.13 28332488

[2] Kordower JH Olanow CW Dodiya HB Chu Y Beach TG Adler CH. Disease duration and the integrity of the nigrostriatal system in Parkinson’s disease. Brain. (2013) 136:2419–31. doi: 10.1093/brain/awt192, PMID: 23884810 PMC3722357

[3] Armstrong MJ Okun MS. Diagnosis and treatment of Parkinson disease: a review. JAMA. (2020) 323:548. doi: 10.1001/jama.2019.22360 32044947

[4] Greenland JC Williams-Gray CH Barker RA. The clinical heterogeneity of Parkinson’s disease and its therapeutic implications. Eur J Neurosci. (2019) 49:328–38. doi: 10.1111/ejn.14094 30059179

[5] Obeso JA Stamelou M Goetz CG Poewe W Lang AE Weintraub D. Past, present, and future of Parkinson’s disease: a special essay on the 200th anniversary of the shaking palsy: the shaking palsy: past. Present Future Mov Disord. (2017) 32:1264–310. doi: 10.1002/mds.27115, PMID: 28887905 PMC5685546

[6] Jankovic J McDermott M Carter J Gauthier S Goetz C Golbe L. Variable expression of Parkinson’s disease: a base-line analysis of the DAT ATOP cohort. Neurology. (1990) 40:1529–34. doi: 10.1212/WNL.40.10.1529, PMID: 2215943

[7] Nutt JG. Motor subtype in Parkinson’s disease: different disorders or different stages of disease?: motor subtypes of PD. Mov Disord. (2016) 31:957–61. doi: 10.1002/mds.26657 27226220

[8] Selikhova M Williams DR Kempster PA Holton JL Revesz T Lees AJ. A clinico-pathological study of subtypes in Parkinson’s disease. Brain. (2009) 132:2947–57. doi: 10.1093/brain/awp234, PMID: 19759203

[9] Aleksovski D Miljkovic D Bravi D Antonini A. Disease progression in Parkinson subtypes: the PPMI dataset. Neurol Sci. (2018) 39:1971–6. doi: 10.1007/s10072-018-3522-z, PMID: 30109466

[10] Goetz CG Tilley BC Shaftman SR Stebbins GT Fahn S Martinez-Martin P. Movement Disorder Society-sponsored revision of the unified Parkinson’s disease rating scale (MDS-UPDRS): scale presentation and clinimetric testing results: MDS-UPDRS: Clinimetric assessment. Mov Disord. (2008) 23:2129–70. doi: 10.1002/mds.22340, PMID: 19025984

[11] Ren J Hua P Pan C Li Y Zhang L Zhang W. Non-motor symptoms of the postural instability and gait difficulty subtype in De novo Parkinson’s disease patients: a cross-sectional study in a single center. Neuropsychiatr Dis Treat. (2020) 16:2605–12. doi: 10.2147/NDT.S280960, PMID: 33173298 PMC7646450

[12] Burn DJ. Motor subtype and cognitive decline in Parkinson’s disease, Parkinson’s disease with dementia, and dementia with Lewy bodies. J Neurol Neurosurg Psychiatry. (2006) 77:585–9. doi: 10.1136/jnnp.2005.081711, PMID: 16614017 PMC2117449

[13] Arie L Herman T Shema-Shiratzky S Giladi N Hausdorff JM. Do cognition and other non-motor symptoms decline similarly among patients with Parkinson’s disease motor subtypes? Findings from a 5-year prospective study. J Neurol. (2017) 264:2149–57. doi: 10.1007/s00415-017-8605-x, PMID: 28879438

[14] Malek N Lawton MA Grosset KA Bajaj N Barker RA Burn DJ. Autonomic dysfunction in early Parkinson’s disease: results from the United Kingdom tracking Parkinson’s study. Mov Disord Clin Pract. (2017) 4:509–16. doi: 10.1002/mdc3.12454, PMID: 30363477 PMC6174464

[15] Wang JY Wang MY Liu RP Li Y Zhang WY Ovlyakulov B. Association analyses of autonomic dysfunction and sympathetic skin response in motor subtypes of Parkinson’s disease. Front Neurol. (2020) 11:577128. doi: 10.3389/fneur.2020.577128, PMID: 33224091 PMC7669620

[16] Jankovic J Kapadia AS. Functional decline in Parkinson disease. Arch Neurol. (2001) 58:1611. doi: 10.1001/archneur.58.10.1611 11594919

[17] Lehericy S Vaillancourt DE Seppi K Monchi O Rektorova I Antonini A. The role of high-field magnetic resonance imaging in parkinsonian disorders: pushing the boundaries forward: MRI in parkinsonian disorders. Mov Disord. (2017) 32:510–25. doi: 10.1002/mds.26968, PMID: 28370449

[18] Brooks DJ. Imaging approaches to Parkinson disease. J Nucl Med. (2010) 51:596–609. doi: 10.2967/jnumed.108.059998 20351351

[19] Rosenberg-Katz K Herman T Jacob Y Kliper E Giladi N Hausdorff JM. Subcortical volumes differ in Parkinson’s disease motor subtypes: new insights into the pathophysiology of disparate symptoms. Front Hum Neurosci. (2016) 10:356. doi: 10.3389/fnhum.2016.00356/abstract, PMID: 27462214 PMC4939290

[20] Rosenberg-Katz K Herman T Jacob Y Giladi N Hendler T Hausdorff JM. Gray matter atrophy distinguishes between Parkinson disease motor subtypes. Neurology. (2013) 80:1476–84. doi: 10.1212/WNL.0b013e31828cfaa4, PMID: 23516323 PMC3662357

[21] Mitchell T Lehéricy S Chiu SY Strafella AP Stoessl AJ Vaillancourt DE. Emerging neuroimaging biomarkers across disease stage in Parkinson disease: a review. JAMA Neurol. (2021) 78:1262–72. doi: 10.1001/jamaneurol.2021.1312, PMID: 34459865 PMC9017381

[22] Pyatigorskaya N Gallea C Garcia-Lorenzo D Vidailhet M Lehericy S. A review of the use of magnetic resonance imaging in Parkinson’s disease. Ther Adv Neurol Disord. (2014) 7:206–20. doi: 10.1177/1756285613511507, PMID: 25002908 PMC4082302

[23] Pasternak O Sochen N Gur Y Intrator N Assaf Y. Free water elimination and mapping from diffusion MRI. Magn Reson Med. (2009) 62:717–30. doi: 10.1002/mrm.22055, PMID: 19623619

[24] Bergmann Ø Henriques R Westin C Pasternak O. Fast and accurate initialization of the free-water imaging model parameters from multi-shell diffusion MRI. NMR Biomed. (2020) 33:e4219. doi: 10.1002/nbm.4219, PMID: 31856383 PMC7110532

[25] Andica K Hatano S Uchida O. Free-water imaging in white and Gray matter in Parkinson’s disease. Cells. (2019) 8:839. doi: 10.3390/cells8080839, PMID: 31387313 PMC6721691

[26] Ofori E Pasternak O Planetta PJ Burciu R Snyder A Febo M. Increased free water in the substantia nigra of Parkinson’s disease: a single-site and multi-site study. Neurobiol Aging. (2015) 36:1097–104. doi: 10.1016/j.neurobiolaging.2014.10.029, PMID: 25467638 PMC4315708

[27] Burciu RG Ofori E Archer DB Wu SS Pasternak O McFarland NR. Progression marker of Parkinson’s disease: a 4-year multi-site imaging study. Brain. (2017) 140:2183–92. doi: 10.1093/brain/awx146, PMID: 28899020 PMC6057495

[28] Planetta PJ Ofori E Pasternak O Burciu RG Shukla P DeSimone JC. Free-water imaging in Parkinson’s disease and atypical parkinsonism. Brain. (2016) 139:495–508. doi: 10.1093/brain/awv361, PMID: 26705348 PMC5790142

[29] Yang J Archer DB Burciu RG Müller MLTM Roy A Ofori E. Multimodal dopaminergic and free-water imaging in Parkinson’s disease. Parkinsonism Relat Disord. (2019) 62:10–5. doi: 10.1016/j.parkreldis.2019.01.007, PMID: 30639168 PMC6589363

[30] Archer DB Bricker JT Chu WT Burciu RG McCracken JL Lai S. Development and validation of the automated imaging differentiation in parkinsonism (AID-P): a multicentre machine learning study. Lancet Digit Health. (2019) 1:e222–31. doi: 10.1016/S2589-7500(19)30105-0, PMID: 33323270

[31] Iacono RP Kuniyoshi SM Ahlman JR Zimmerman GJ Maeda G Pearlstein RD. Concentrations of indoleamine metabolic intermediates in the ventricular cerebrospinal fluid of advanced Parkinson’s patients with severe postural instability and gait disorders. J Neural Transm. (1997) 104:451–9. doi: 10.1007/BF01277663, PMID: 9295177

[32] Mohl B Berman BD Shelton E Tanabe J. Levodopa response differs in Parkinson’s motor subtypes: a task-based effective connectivity study. J Comp Neurol. (2017) 525:2192–201. doi: 10.1002/cne.24197, PMID: 28256710 PMC6301039

[33] Vu TC Nutt JG Holford NHG. Progression of motor and nonmotor features of Parkinson’s disease and their response to treatment: progression of Parkinson’s disease. Br J Clin Pharmacol. (2012) 74:267–83. doi: 10.1111/j.1365-2125.2012.04192.x, PMID: 22283961 PMC3630747

[34] Marek K Jennings D Lasch S Siderowf A Tanner C Simuni T. The Parkinson progression marker initiative (PPMI). Prog Neurobiol. (2011) 95:629–35. doi: 10.1016/j.pneurobio.2011.09.005, PMID: 21930184 PMC9014725

[35] Marek K Chowdhury S Siderowf A Lasch S Coffey CS Caspell-Garcia C. The Parkinson’s progression markers initiative (PPMI) – establishing a PD biomarker cohort. Ann Clin Transl Neurol. (2018) 5:1460–77. doi: 10.1002/acn3.644, PMID: 30564614 PMC6292383

[36] Postuma RB Berg D Stern M Poewe W Olanow CW Oertel W. MDS clinical diagnostic criteria for Parkinson’s disease: MDS-PD Clinical Diagnostic Criteria. Mov Disord. (2015) 30:1591–601. doi: 10.1002/mds.26424 26474316

[37] Nasreddine ZS Phillips NA Charbonneau S Whitehead V Collin I. The Montreal cognitive assessment, MoCA: a Brief Screening Tool for mild cognitive impairment: MOCA: a BRIEF SCREENING TOOL FOR MCI. J Am Geriatr Soc. (2005) 53:695–9. doi: 10.1111/j.1532-5415.2005.53221.x, PMID: 15817019

[38] Mitchell T Wilkes BJ Archer DB Chu WT Coombes SA Lai S. Advanced diffusion imaging to track progression in Parkinson’s disease, multiple system atrophy, and progressive supranuclear palsy. Neuro Image Clin. (2022) 34:103022. doi: 10.1016/j.nicl.2022.103022 PMC9062732 35489192

[39] Mitchell T Archer DB Chu WT Coombes SA Lai S Wilkes BJ. Neurite orientation dispersion and density imaging (NODDI) and free-water imaging in parkinsonism. Hum Brain Mapp. (2019) 40:5094–107. doi: 10.1002/hbm.24760, PMID: 31403737 PMC6865390

[40] Avants BB Tustison NJ Song G Cook PA Klein A Gee JC. A reproducible evaluation of ANTs similarity metric performance in brain image registration. Neuro Image. (2011) 54:2033–44. doi: 10.1016/j.neuroimage.2010.09.025, PMID: 20851191 PMC3065962

[41] Kim SH Kim JS. Isolated infarction of anterior cerebellar vermis. Res Vestib Sci. (2016) 15:147–50. doi: 10.21790/rvs.2016.15.4.147

[42] Ilg W Giese MA Gizewski ER Schoch B Timmann D. The influence of focal cerebellar lesions on the control and adaptation of gait. Brain. (2008) 131:2913–27. doi: 10.1093/brain/awn246, PMID: 18835866

[43] Palakurthi B Burugupally SP. Postural instability in Parkinson’s disease: a review. Brain Sci. (2019) 9:239. doi: 10.3390/brainsci9090239, PMID: 31540441 PMC6770017

[44] Skidmore FM Monroe WS Hurt CP Nicholas AP Gerstenecker A Anthony T. The emerging postural instability phenotype in idiopathic Parkinson disease. Npj Park Dis. (2022) 8:28. doi: 10.1038/s41531-022-00287-x, PMID: 35304493 PMC8933561

[45] Alexander GE Crutcher MD. Functional architecture of basal ganglia circuits: neural substrates of parallel processing. Trends Neurosci. (1990) 13:266–71. doi: 10.1016/0166-2236(90)90107-L, PMID: 1695401

[46] Lanciego JL Luquin N Obeso JA. Functional neuroanatomy of the basal ganglia. Cold Spring Harb Perspect Med. (2012) 2:a009621. doi: 10.1101/cshperspect.a009621, PMID: 23071379 PMC3543080

[47] Obeso JA Rodríguez-Oroz MC Rodríguez M Arbizu J Giménez-Amaya JM. The basal ganglia and disorders of movement: pathophysiological mechanisms. Physiology. (2002) 17:51–5. doi: 10.1152/nips.01363.2001 11909992

[48] Obeso JA Rodriguez-Oroz MC Rodriguez M Lanciego JL Artieda J Gonzalo N. Pathophysiology of the basal ganglia in Parkinson’s disease. Trends Neurosci. (2000) 23:S8–S19. doi: 10.1016/S1471-1931(00)00028-8 11052215

[49] Blandini F Nappi G Tassorelli C Martignoni E. Functional changes of the basal ganglia circuitry in Parkinson’s disease. Prog Neurobiol. (2000) 62:63–88. doi: 10.1016/S0301-0082(99)00067-2 10821982

[50] Lee JW Song YS Kim H Ku BD Lee WW. Alteration of tremor dominant and postural instability gait difficulty subtypes during the progression of Parkinson’s disease: analysis of the PPMI cohort. Front Neurol. (2019) 10:471. doi: 10.3389/fneur.2019.00471, PMID: 31133973 PMC6514149

[51] Nagae LM Honce JM Tanabe J Shelton E Sillau SH Berman BD. Microstructural changes within the basal ganglia differ between Parkinson disease subtypes. Front Neuroanat. (2016) 10:17. doi: 10.3389/fnana.2016.00017/abstract, PMID: 26941615 PMC4763054

[52] Mirdamadi JL. Cerebellar role in Parkinson’s disease. J Neurophysiol. (2016) 116:917–9. doi: 10.1152/jn.01132.2015, PMID: 26792889 PMC5009206

[53] Wu T Hallett M. The cerebellum in Parkinson’s disease. Brain. (2013) 136:696–709. doi: 10.1093/brain/aws360, PMID: 23404337 PMC7273201

[54] Solstrand Dahlberg L Lungu O Doyon J. Cerebellar contribution to motor and non-motor functions in Parkinson’s disease: a meta-analysis of fMRI findings. Front Neurol. (2020) 11:127. doi: 10.3389/fneur.2020.00127, PMID: 32174883 PMC7056869

[55] Bostan AC Dum RP Strick PL. Cerebellar networks with the cerebral cortex and basal ganglia. Trends Cogn Sci. (2013) 17:241–54. doi: 10.1016/j.tics.2013.03.003, PMID: 23579055 PMC3645327

[56] Middleton FA Strick PL. Cerebellar projections to the prefrontal cortex of the primate. J Neurosci. (2001) 21:700–12. doi: 10.1523/JNEUROSCI.21-02-00700.2001, PMID: 11160449 PMC6763818

[57] O’Callaghan C Hornberger M Balsters JH Halliday GM Lewis SJG Shine JM. Cerebellar atrophy in Parkinson’s disease and its implication for network connectivity. Brain. (2016) 139:845–55. doi: 10.1093/brain/awv399 26794597

[58] Uddin MN Faiyaz A Wang L Zhuang Y Murray KD Descoteaux M. A longitudinal analysis of brain extracellular free water in HIV infected individuals. Sci Rep. (2021) 11:8273. doi: 10.1038/s41598-021-87801-y, PMID: 33859326 PMC8050285

[59] Simuni T Caspell-Garcia C Coffey C Lasch S Tanner C Marek K. How stable are Parkinson’s disease subtypes in de novo patients: analysis of the PPMI cohort? Parkinsonism Relat Disord. (2016) 28:62–7. doi: 10.1016/j.parkreldis.2016.04.027, PMID: 27132498

[60] Guttuso T Bergsland N Hagemeier J Lichter DG Pasternak O Zivadinov R. Substantia Nigra free water increases longitudinally in Parkinson disease. Am J Neuroradiol. (2018) 39:479–84. doi: 10.3174/ajnr.A5545, PMID: 29419398 PMC6070442

[61] Von Coelln R Gruber-Baldini AL Reich SG Armstrong MJ Savitt JM Shulman LM. The inconsistency and instability of Parkinson’s disease motor subtypes. Parkinsonism Relat Disord. (2021) 88:13–8. doi: 10.1016/j.parkreldis.2021.05.016, PMID: 34091412

